# Bovine serum albumin sensitizes the mouse brain to lipopolysaccharides

**DOI:** 10.1371/journal.pone.0356781

**Published:** 2026-08-26

**Authors:** Yan Jiang, Yuhu Feng, Xinyu Li, Bing Zhao, Zuisu Yang, Ruling Wei, Falei Yuan

**Affiliations:** 1 Department of Pharmacy, School of Food and Pharmacy, Zhejiang Ocean University, Zhoushan, China; 2 Department of Physical Education, Zhejiang Ocean University, Zhoushan, China; 3 Department of Emergency, Ruijin Hospital, Shanghai Jiao Tong University School of Medicine, Shanghai, China; Noorda College of Osteopathic Medicine, UNITED STATES OF AMERICA

## Abstract

This study examined the role of bovine serum albumin (BSA) in lipopolysaccharide (LPS)-induced brain injury. Mice were intraperitoneally injected with either LPS or LPS coupled with BSA. Brain tissues were analyzed for autofluorescence, vascular cell permeability, blood-brain barrier integrity, and pyroptosis. Co-immunoprecipitation and immunostaining were performed to assess the interaction between albumin and LPS. The results showed that, compared to the 5 mg/kg LPS group, the 5 mg/kg BSA-LPS group exhibited enhanced autofluorescence and an increased number of cells with co-localization of propidium iodide (PI) with cluster of differentiation 31 (CD31) and CD13. However, no significant differences were observed between the 25 mg/kg LPS and 25 mg/kg BSA-LPS groups. Horseradish peroxidase (HRP) labeling revealed higher HRP leakage in the 5 mg/kg BSA-LPS group compared to the 5 mg/kg LPS group, a difference that disappeared at the 25 mg/kg dose. Peroxidase staining showed a similar trend. Immunostaining for gasdermin D and CD11b showed significant differences only between the 5 mg/kg LPS and 5 mg/kg BSA-LPS groups. CD68 immunostaining exhibited a similar trend. Furthermore, the study confirmed that albumin binds to LPS in the brain, with more abundant Lipid A^+^ signals detected in the 5 mg/kg BSA-LPS group compared to the 5 mg/kg LPS group. These findings collectively suggest that albumin boosts the effect of LPS in the brain, probably by binding with LPS and increasing its entry into the brain parenchyma.

## 1. Introduction

Sepsis is a severe condition caused by a dysregulated host response to infection [1]. Despite continuous improvements in medical conditions, sepsis-related mortality remains high [2]. Globally, sepsis accounted for 11 million deaths, which corresponded to 19.7% of total mortality [3]. Studies indicate that sepsis-associated encephalopathy (SAE) occurs in approximately 70% of sepsis patients, which involves blood-brain barrier (BBB) dysfunction, neurotransmitter alterations, and neuroinflammation [4]. Although the exact mechanisms for inducing SAE are still unknown, pyroptosis-associated pathways have been recognized as an important factor in the pathogenesis of SAE [5].

Gram-negative bacteria are the most prevalent pathogens identified in sepsis [6,7]. A key player in Gram-negative sepsis is lipopolysaccharide (LPS), a major component of the bacterial cell wall [8]. The architecture of LPS comprises three main regions: the hydrophobic Lipid A, the core oligosaccharide, and the variable O-antigen chain. LPS exerts its effects by stimulating the transmembrane TLR4 receptor to activate innate immunity [9], and by binding intracellularly to caspase-11 to induce pyroptosis [10]. Therefore, LPS is frequently used to establish sepsis models in mice [11].

Fluid resuscitation has been a standard intervention for combating sepsis, involving the intravenous administration of crystalloids and colloids. Early fluid administration in sepsis can improve microcirculatory blood flow and increase cardiac output [12]. Albumin is considered the most important type of colloids for treating sepsis, as it is an endogenous protein that contributes to homeostasis and fatty acid transport [13]. Also, 30% to 50% of critically ill patients exhibit hypoalbuminemia (albumin concentration <35 g/L), a condition associated with higher mortality [14]. Both low- and high-concentration human serum albumin solutions are commonly used for fluid resuscitation in septic patients [15]. Studies have confirmed the safety of 4% [16] and 20% [17] albumin solutions in sepsis treatment. Albumin has been reported to neutralize LPS, thereby preventing its toxic effects [18]. However, some literature reports that albumin administration in sepsis may worsen the condition [19] or increase net albumin leakage [20]. Beyond its clinical application, albumin is essential for cognitive health, as evidenced by its significantly lower levels in Alzheimer's disease (AD) patients compared to healthy controls [21–23] and its association with poor cognition performance [24]. However, evidence does not support albumin administration for reducing mortality in critically ill patients with hypovolemia, burns, or hypoalbuminemia, and it may even increase mortality risk [25]. Albumin is only recommended after receiving a large volume of crystalloids in the 2021 international guidelines for management of sepsis and septic shock, as it is more expensive and shows no significant benefit [26].

The bioactivity of LPS is primarily mediated by its hydrophobic Lipid A component, which is insoluble in aqueous environments [27]. In enzyme-linked immunosorbent assays (ELISA) experiments, when LPS and its complex with bovine serum albumin (BSA-LPS) are separately coated onto microplates, the BSA-LPS group demonstrates superior coating homogeneity [28]. In vivo experiments show that mice administered with the BSA-LPS combination exhibit a higher production of inflammatory cytokines compared to those given LPS alone [29]. Our previous study finds that, compared to the palmitic acid group and BSA group, the complex formed between palmitic acid and BSA increases the intracellular LPS content [30]. Clinically, therapeutic plasma exchange with albumin replacement emerges as a promising treatment for sepsis [31]. This study aims to investigate whether incubating LPS with BSA enhances its effects in the mouse brain, which may help us understand the role of albumin in patients with sepsis.

## 2. Materials and methods

### 2.1. Animals

Six-week-old male ICR mice were purchased from Hangzhou Muhao Inc. (Zhejiang, China). The animal studies were approved by the Experimental Animal Ethics Committee of Zhejiang Ocean University (Approval Numbers: SCXK 2019−0031, #2025059).

### 2.2. LPS administration

A bovine serum albumin (BSA, #AS33654, LANSO) solution was prepared by dissolving 5.5 mg of BSA powder in 2.5 mL of normal saline. For the BSA-LPS complex, 7.5 mg of LPS derived from Escherichia coli O55:B5 (#ST1470, Beyotime) was added to the prepared BSA solution and incubated in a water bath at 37 °C for 30 min. Subsequently, the mice were administered LPS or the BSA-LPS complex via intraperitoneal injection at doses of 5 mg/kg and 25 mg/kg, with corresponding injection volumes of 50 µL or 250 µL per mouse.

### 2.3. Autofluorescence (AF) detection, PI labeling, and immunofluorescence staining

Mouse brain tissues were collected from mice sacrificed with carbon dioxide and perfused transcardially with PBS followed by 4% paraformaldehyde (PFA). The extracted brains were then embedded in low-melting-point agarose and sectioned into 50 µm-thick slices using a vibratome (ZQP-86, Zhisun Equipment Inc., Shanghai). For AF detection, the sections were stained with 4,’6-diamidino-2-phenylindole (DAPI) and the barrel cortex was visualized using the rhodamine channel with an excitation wavelength of 450–490 nm. For PI labeling, the mice received an intravenous tail vein injection of PI (#P113815, Aladdin, 10 mg/kg, 50 µL per mouse) 24 h after the LPS injection. For immunofluorescence detection, the sections were first incubated with 0.3% Triton X-100 and 1% BSA for 15 min. They were then incubated with the following primary antibodies at a 1:400 dilution for 2 h at room temperature: cluster of differentiation 31 (CD31, #550274, BD Biosciences) for endothelial cells, cluster of differentiation 13 (CD13, #GTX75927, Genetex) for pericytes, gasdermin-D (GSDMD, #ab209845, Abcam) for apoptosis, cluster of differentiation 11b (CD11b, #557394, BD Biosciences) for microglia, cluster of differentiation 68 (CD68, # MCA1957, Bio-Rad Laboratories) for activated microglia, and Lipid A (#ab8467, Abcam) for LPS. Subsequently, the sections were incubated with secondary antibodies (#111-095-003, #115-095-003, #112-095-003, Jackson ImmunoResearch; #A11010, Thermo Fisher Scientific) for 1 h at room temperature. Finally, the sections were stained with DAPI and visualized in the cortex region or the barrel cortex region using the rhodamine channel with an excitation wavelength of 450–490 nm or the Fluorescein Isothiocyanate channel with an excitation wavelength of 488–546 nm. Images were acquired using an Olympus BX41 microscope equipped with a digital camera (Olympus DP26). Images were captured with the same filter (U‑25ND6) and a manually set exposure time (300 ms).

Image analysis was performed using ImageJ software (version 1.54, National Institutes of Health). For quantitative analysis of AF, fluorescent images were first converted to 8-bit format, followed by manual adjustment of the threshold range to encompass all visible fluorescent signals. The area fraction (%Area) occupied by fluorescence was then measured using the measurement function. For immunofluorescence staining, quantification was performed by calculating the ratio of positive cells to total cells (DAPI). For double immunofluorescence staining, quantification was performed by calculating the ratio of colocalized positive cells to single-positive cells.

Unless otherwise stated, each data point represented the positive signal value obtained from a randomly selected section from the brain slices of each mouse according to the analysis procedures described above. For mice injected with PI via the tail vein, the scanned region was the cortical area of the section, whereas for the remaining slices, the scanned region was the barrel cortex area. The experimental operations were performed by Yan Jiang, and the data analysis was conducted by Yuhu Feng.

### 2.4. BBB leakage detection

For horseradish peroxidase (HRP) labeling of BBB leakage, a 20 mg/mL solution of HRP (#P815746, MACKLIN) was prepared in normal saline. Then, 50 µL of this solution was administered to mice via tail vein injection 24 h after the LPS injection. After circulating for 10 min, the mice were transcardially perfused with PBS. Subsequently, 3,3’-diaminobenzidine (DAB) staining was performed to detect the leaked HRP. For immunoglobulin G (IgG) staining, brain sections were pre-heated at 90 °C for 30 min to quench endogenous peroxidase [32]. HRP-DAB staining was performed using an IHC detection kit (#PK10006, Proteintech). For endogenous peroxidase detection, HRP-DAB staining was carried out directly using the IHC detection kit.

For the assessment of BBB leakage, imaged of the cortex or barrel cortex were acquired using an Olympus CX31 microscope equipped with a 20 × objective (Olympus Plan C N, NA 0.6) and Pro-MicroScan NC6050 digital camera. For quantitative analysis, image analysis was performed using ImageJ software (version 1.54, National Institutes of Health), with reference to the literature of Raheleh [33] and Anthony [34]. Images were first subjected to color deconvolution. Subsequently, the DAB channel was manually thresholded to include all visible signals, and the positive area was measured to calculate the Optical density (OD)×%Positive Cells value, where the OD value was calculated as log (255/MEAN).

Each data point represents the statistical value obtained from staining a randomly selected section of the brain slice from each mouse. The experimental operations were performed by Yuhu Feng, and the data analysis was conducted by Yan Jiang.

### 2.5. Co-Immunoprecipitation (Co-IP) assay

Mouse brain tissues were weighed (approximately 50 mg) and processed with a Co-IP kit according to the manufacturer’s instructions (#P2179S, Beyotime). Briefly, brain tissues were lysed and centrifuged at 12,000 rpm for 10 min. The supernatant was mixed with Protein A/G magnetic beads that were pre-incubated with the following antibodies: albumin (#ab207327, Abcam), LPS Core (#HM6011, Hycult Biotech), and Immunoglobulin G antibody (IgG, #B900620, Proteintech). For immunoblotting, primary antibodies LPS Core (#HM6011, Hycult Biotech) and albumin (#ab207327, Abcam), and secondary antibodies (#HA1006, #HA1001, HUABIO) were used.

### 2.6. Software

All statistical analyses were performed using GraphPad Prism (version 9.0). Data were expressed as mean ± SEM. To evaluate differences across multiple groups, we employed a one-way analysis of variance (ANOVA), with post-hoc comparisons performed using Tukey’s test. For analyzing two-group data, an unpaired t-test (independent samples t-test) was applied. A *p*-value below 0.05 was assigned statistically significance.

## 3. Results

### 3.1. BSA coupling enhances the effects of LPS in inducing AF

**Fig 1:**
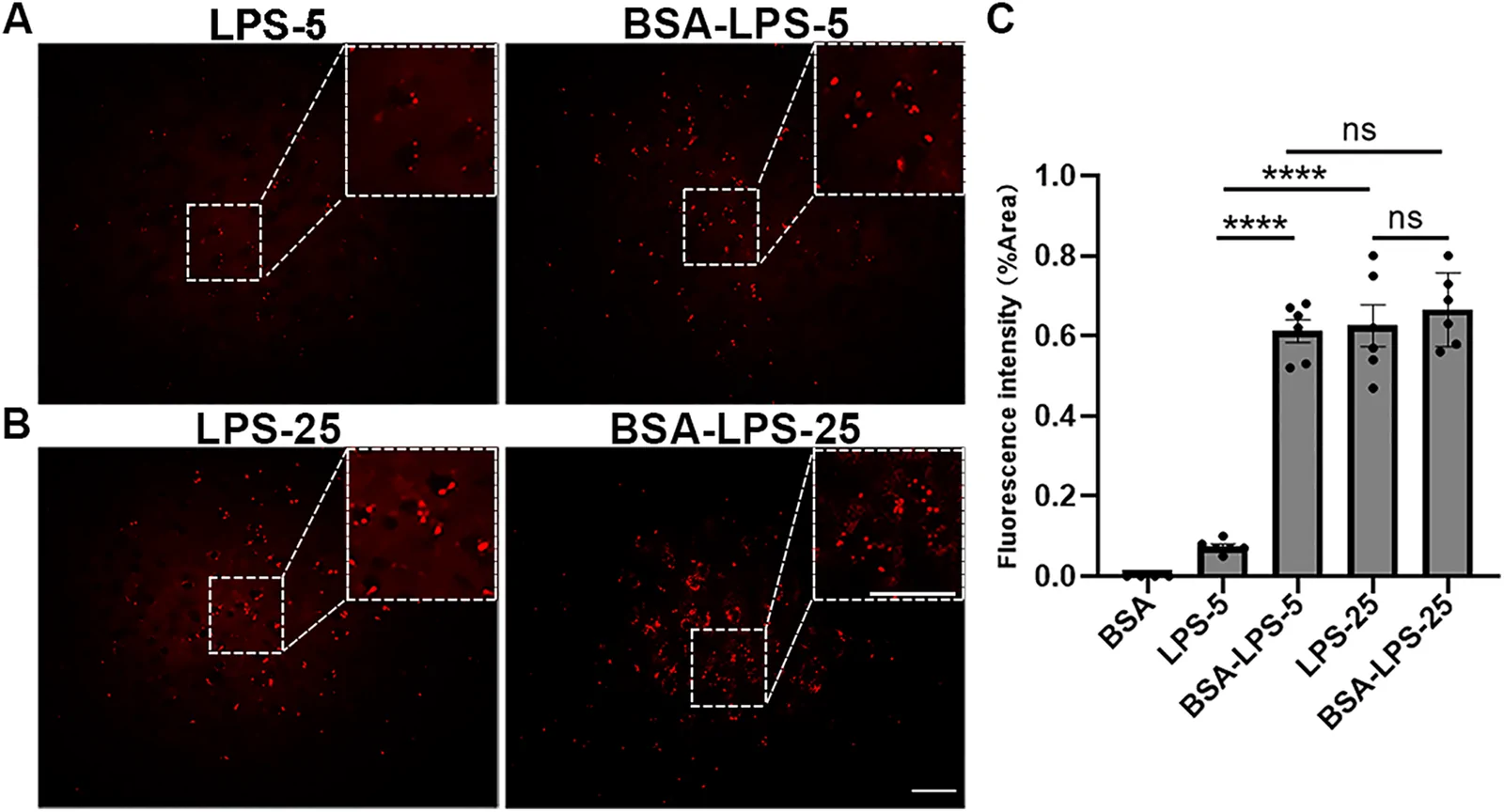
Effects of BSA coupling with LPS on AF induction. (A, B) Representative images of mouse brain sections of the four groups (n = 6). (C) Statistical analysis of AF levels induced by different doses of LPS or LPS incubated with BSA (n = 6 for each LPS-treated group, n = 4 for the BSA-only group). Significance was analyzed by one-way ANOVA with Tukey’s multiple comparisons test. Scale bar: 20 µm. ns, not significant; ****p < 0.0001.

AF is one of the characteristic features of lipofuscin, which is a hallmark of aging. Our previous study found that LPS induces AF in the mouse brain, a phenomenon that resembles natural aging [35]. To investigate the effect of BSA on LPS action, we intraperitoneally administered different doses of LPS, either alone or in combination with BSA (BSA-LPS), to the mice. The mice were divided into five groups: the BSA group (n = 4), the 5 mg/kg LPS (LPS-5) group (n = 6), the 5 mg/kg BSA-LPS (BSA-LPS-5) group (n = 6), the 25 mg/kg LPS (LPS-25) group (n = 6), and the 25 mg/kg BSA-LPS (BSA-LPS-25) group (n = 6). Mouse brains were harvested 2 weeks after LPS injection and examined directly under a fluorescent microscope. The results showed that there was no AF-positive signal in the BSA group; the AF level in the BSA-LPS-5 group was significantly higher than that in the LPS-5 group (Fig 1A, C, S1). The LPS-25 group exhibited significantly higher AF than the LPS-5 group (Fig 1A-C). However, AF in the BSA-LPS-25 group showed no significant difference in AF induction compared to the LPS-25 group or the BSA-LPS-5 group (Fig 1A-C). These results indicate that BSA enhances AF under low-dose LPS treatment in the mouse brain.

### 3.2. BSA enhances LPS-induced vascular cell permeability

**Fig 2:**
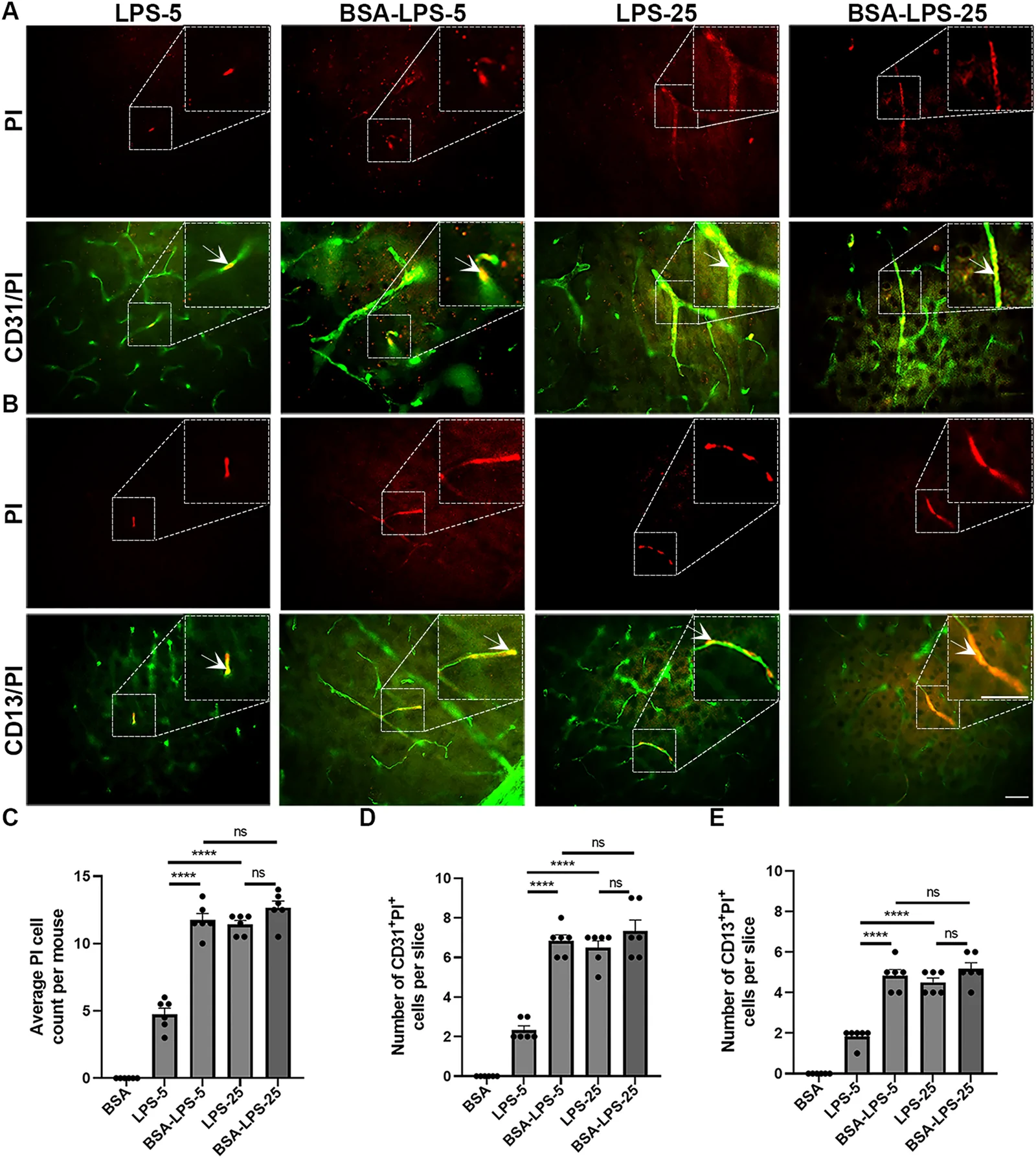
Effects of BSA coupling with LPS on vascular cell permeability. (A, B) Immunostaining for CD31 and CD13 of the four groups after tail vein injection of PI (n = 6). The PI images and the corresponding CD31/PI and CD13/PI fluorescence staining results were captured in the same field of view different channels of the fluorescence microscope. (C) Quantitative analysis was performed on two brain sections per mouse, and the average number of PI^+^ cells was used for statistical analysis. (D, E) Quantitative analysis of the number of PI^+^ cells co-localized with CD31 and CD13. Significance was analyzed by one-way ANOVA with Tukey’s multiple comparisons test. Arrows indicate PI^+^CD31^+^ or PI^+^CD13^+^ cells. Scale bar: 20 µm. ns, not significant; ****p < 0.0001.

To further understand the effects of BSA supplementation on LPS effects on cell permeability, we injected PI through the tail vein 24 h after LPS treatment and immunostained brain sections for CD31 and CD13 (n = 6). The results indicate that the number of PI^+^ cells in the BSA-LPS-5 group was significantly greater than that in the LPS-5 group (Fig 2A-C, S2). Furthermore, the numbers of CD31^+^PI^+^ cells and CD13^+^PI^+^ cells in the BSA-LPS-5 group were significantly greater than that in the LPS-5 group (Fig 2A, B, D, E). However, no significant difference was observed in the number of PI^+^ cells between the BSA-LPS-25 group and either the LPS-25 group or the BSA-LPS-5 group (Fig 2A-E). In addition, we found that the number of PI^+^ cells was approximately twice that of CD31^+^ or CD13^+^ cells. These results indicate that BSA significantly enhances the effects of LPS-induced cell permeability in either endothelial cells or pericytes.

### 3.3. BSA can enhance the effect of LPS on BBB leakage

**Fig 3:**
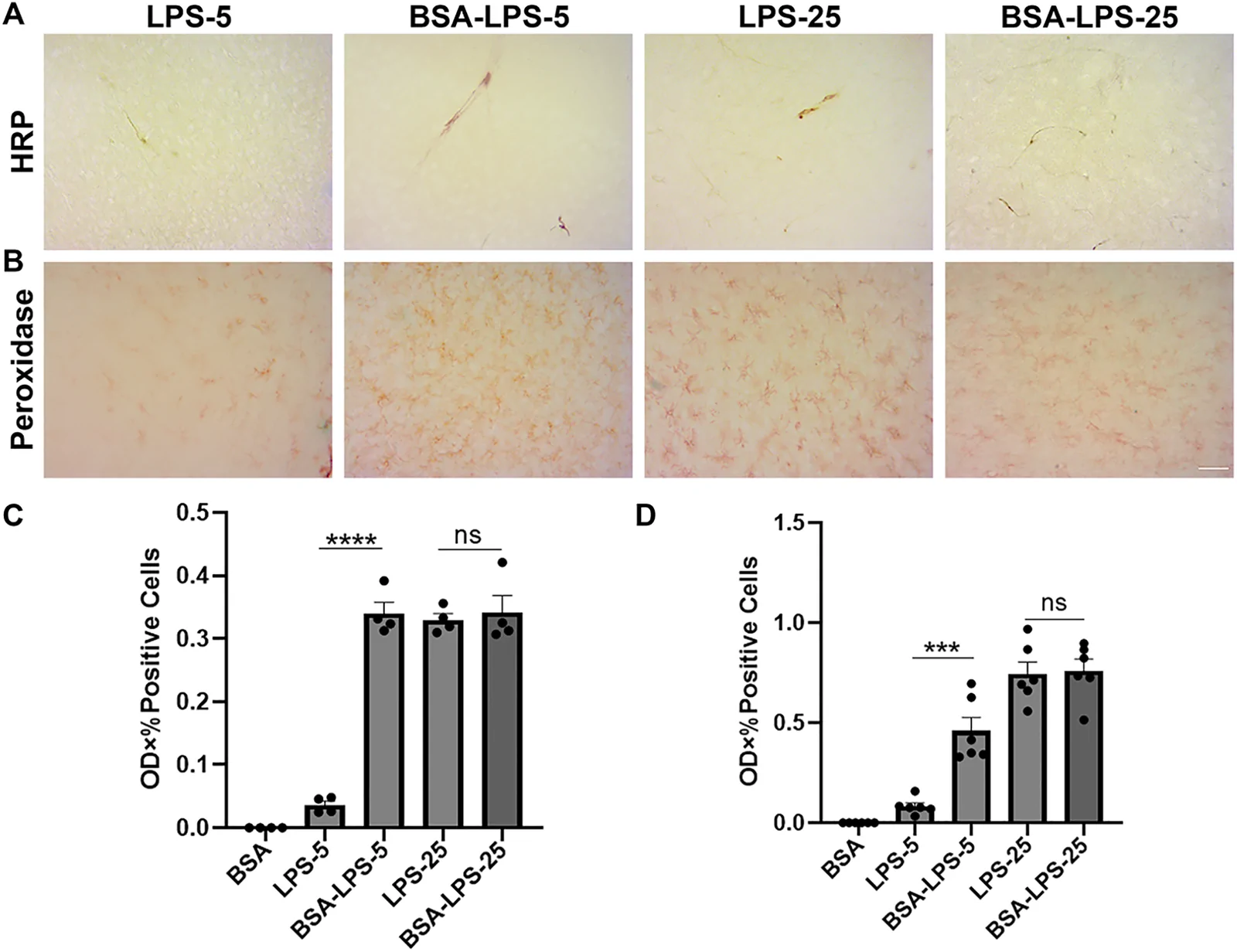
Effects of BSA binding with LPS on BBB integrity. (A) HRP labeling results for the four groups 24 h after LPS administration (n = 4). (B) Direct HRP-DAB staining for endogenous peroxidase (n = 6). (C) OD × %Positive Cells of HRP-positive areas in the four groups. (D) OD × %Positive Cells of peroxidase-positive areas in the four groups. Significance was determined by an unpaired t-test. Scale bar: 50 µm. ns, not significant; ***p < 0.001; ****p < 0.0001.

To further confirm the effect of BSA on LPS-induced BBB disruption, we injected HRP through the tail vein 24 h after LPS treatment and performed DAB staining on mouse brain sections (n = 4). Microscopic analysis revealed that the OD × %Positive Cells value in the BSA-LPS-5 group was significantly higher than that in the LPS-5 group (Fig 3A, C, S3). However, no significant difference was observed between the BSA-LPS-25 group and the LPS-25 group (Fig 3A, C). The same trend was observed in peroxidase staining (Fig 3B, D). To eliminate background staining, we performed IgG staining on the samples using an optimized protocol (n = 6), in which the samples were heated at 90 °C for 30 min [32]. In contrast, IgG staining with an optimized protocol showed no positive signal in the four groups. These results indicate that BSA significantly enhances the effect of LPS on BBB leakage. The size range for BBB opening is between 44 kDa and 150 kDa.

### 3.4. BSA enhances LPS-induced pyroptosis in microglia

**Fig 4:**
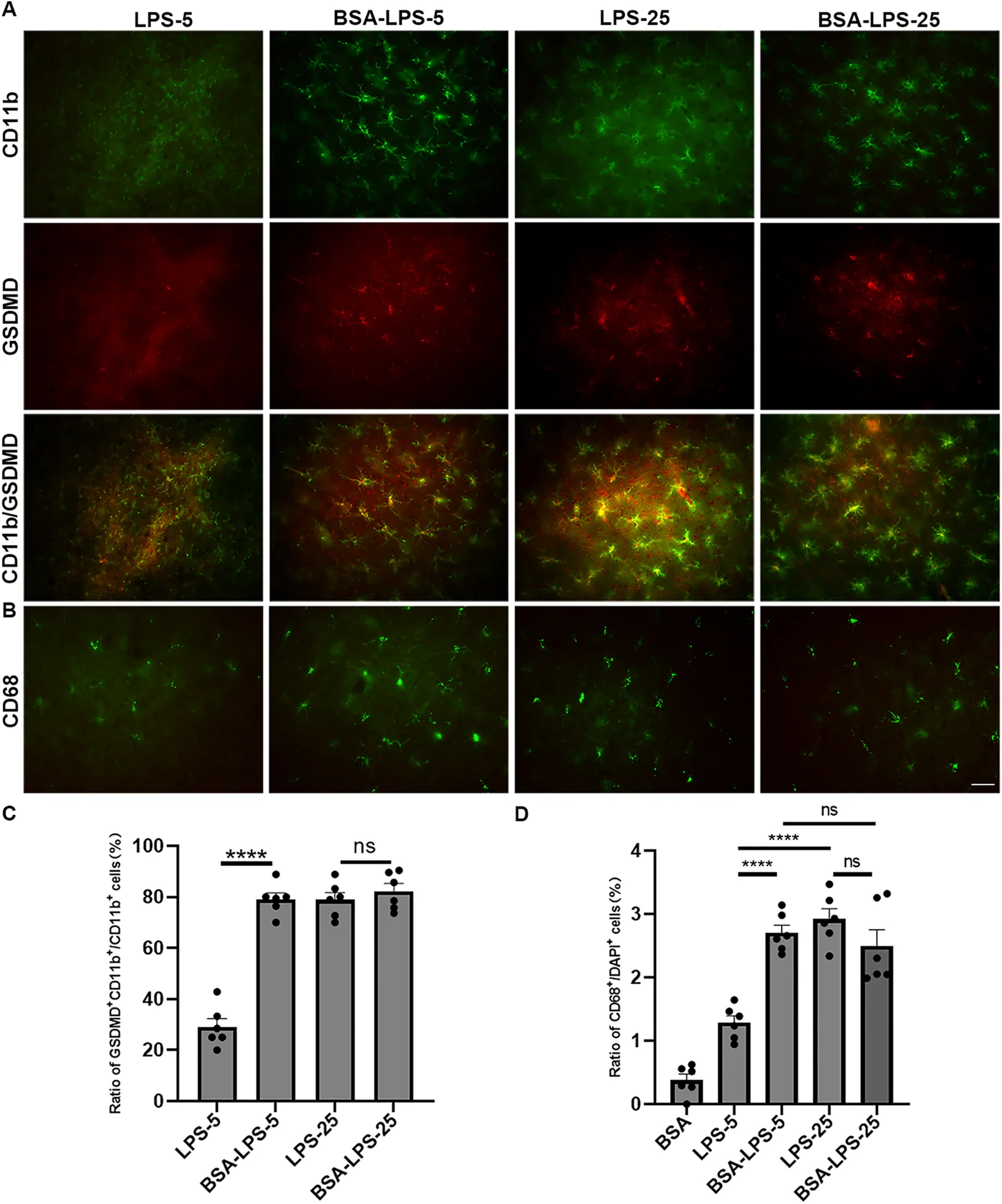
Effects of BSA and LPS co-administration on microglia. (A) Representative immunostaining results for CD11b and GSDMD in the cortex across the four groups (n = 6). (B) Representative immunostaining results for CD68^+^ in the cortex across the groups (n = 6). (C) Proportions of the proportion of GSDMD^+^CD11b^+^ cells in the cortical region. (D) Proportions of the proportion of CD68^+^ cells in the cortical region. Significance was determined using an unpaired t-test. Scale bar: 20 µm. ns, not significant; ****p < 0.0001.

To detect the effect of BSA coupling on other brain cells, we immunostained brain tissue samples after LPS treatment for CD11b and GSDMD (n = 6). The BSA-LPS-5 group exhibited a significant increase in the number of cells with co-localization of CD11b and GSDMD compared to the LPS-5 group (Fig 4A, C). However, the number of cells with CD11b and GSDMD co-localization was similar between the LPS-25 and the BSA-LPS-25 groups (Fig 4A, C). In addition, we further performed immunofluorescence staining for CD68, a marker of microglial activation, on these samples and observed a similar trend: the BSA-LPS-5 group showed a significant increase in the number of CD68^+^ cells compared to the LPS-5 group; however, no significant difference was detected between the LPS-25 and the BSA-LPS-25 groups (Fig 4B, D, S4). These results suggest that BSA significantly enhances LPS-induced microglial activation and pyroptosis.

### 3.5. Albumin in mouse brain parenchyma interacts with LPS

**Fig 5:**
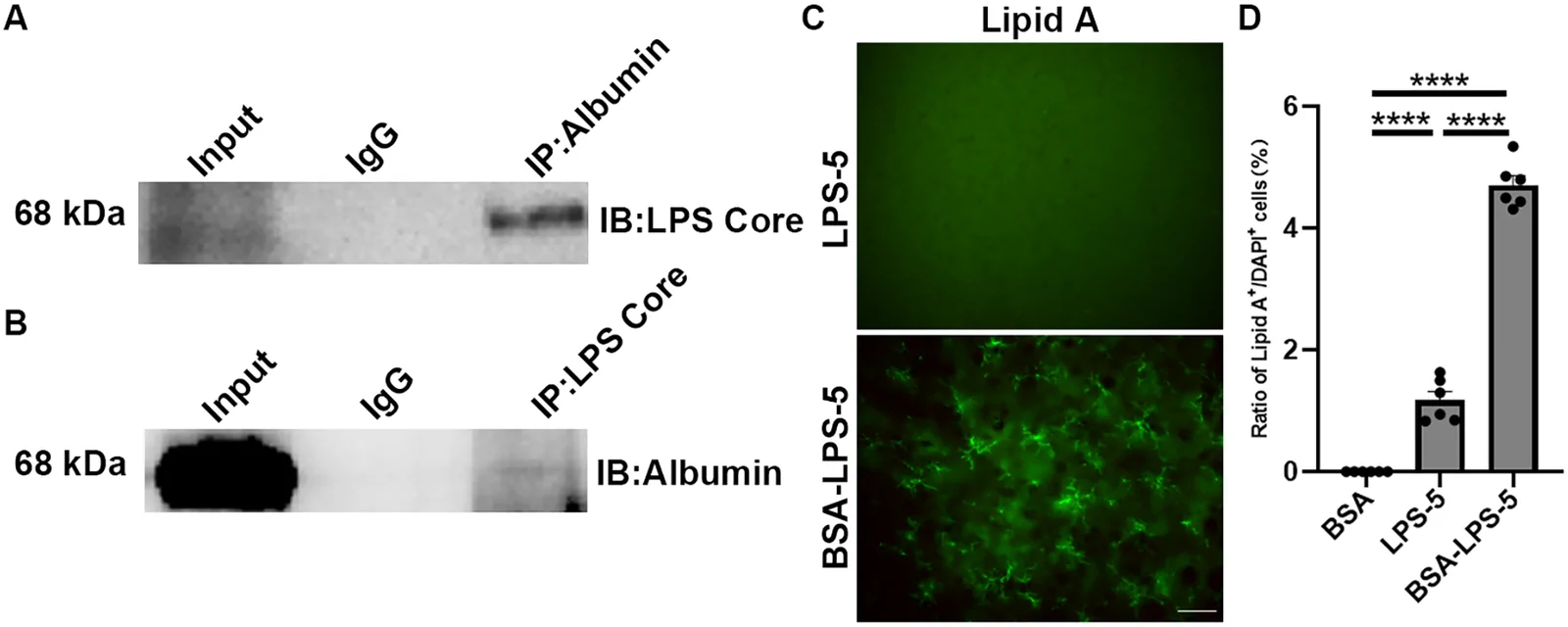
Interaction between albumin and LPS. (A) Co-IP assay using an anti-albumin antibody for IP and an anti-LPS Core for immunoblotting (IB). (B) Co-IP assay using an anti-LPS Core antibody for IP and an anti-albumin antibody for IB. (C) Representative images of Lipid A immunostaining in the LPS-5 group and the BSA-LPS-5 group (n = 6). (D) Proportion of Lipid A^+^ cells in mouse brain sections from the different LPS administration groups. Scale bar: 20 µm. ****p < 0.0001.

To understand the possible mechanisms underlying the effects of BSA coupling, the mouse cortex was subjected to immunoprecipitation with an anti-albumin antibody and immunoblotting with an anti-LPS Core antibody. A clear band was detected near 68 kDa instead of the expected 15 kDa (Fig 5A); this is consistent with results from Vargas-Caraveo et al. that show LPS binds to different molecular weight proteins [36]. Immunoprecipitation with the anti-LPS Core antibody followed by immunoblotting using the anti-albumin antibody further confirms the interaction between LPS Core and albumin (Fig 5B). These results indicate that the LPS molecule binds to albumin in the mouse brain. To determine whether BSA exacerbates LPS-induced neuroinflammation via Lipid A, given that Lipid A is the toxic component of LPS, we performed Lipid A staining on brain sections from mice treated with BSA, LPS-5, and BSA-LPS-5 (n = 6). The results showed significantly higher Lipid A levels in the BSA-LPS-5 group compared to the LPS-5 group (Fig 5C, D), indicating that after entering the brain via LPS Core binding to albumin, LPS still exerts its toxic effects through Lipid A.

## 4. Discussion

Our research reveals that BSA promotes the progression of LPS-induced pathology, specifically by exacerbating AF, vascular hyperpermeability, and microglial pyroptosis. Mechanistically, we discovered that albumin interacts with LPS, and BSA co-administration with LPS significantly increases the entry of LPS into the brain parenchyma.

While previous literature has reported that LPS primarily induces pyroptosis in endothelial cells [37], our study revealed that pyroptosis also occurs in pericytes. This discrepancy is potentially due to differences in LPS administration dosage, as pericytes have been shown to be more susceptible to lesions than microglia [38]. Our previous lineage-tracing study has shown that a single dose of 5 mg/kg LPS without BSA can give rise to vascular remodeling, including the renewal of some pericytes [39]. The dosages of 5 mg/kg and 25 mg/kg were chosen for this study to avoid high mortality caused by high doses of LPS, because the time point we checked was much longer than the 6 h time point used in the study with a higher dosage of LPS (54 mg/kg) [37].

The binding capacity of BSA for fatty acids increases dramatically from a physiological ratio of 1:1–1:2 [40] to a pathological ratio of up to 1:6 [41], or even higher [42]. The molar ratio of BSA to LPS was set at 1:6 to achieve saturation of BSA with LPS. However, the effects of BSA coupling on LPS-induced vascular cell permeability in the BSA-LPS-25 group and the BSA-LPS-5 group are similar, even though the BSA-LPS-25 group contains five times more BSA. As reported, the ability of brain tissue to take up LPS from the peripheral circulation is limited, so the amount of LPS that ultimately enters the brain parenchyma is also limited [43]. In the BSA-LPS-5 group, BSA‑mediated transport of LPS into the brain parenchyma may have reached saturation; therefore, increasing the dose to LPS‑25 or BSA‑LPS‑25 did not further increase the amount of LPS entering the brain parenchyma.

We propose that BSA could leverage this intrinsic transport function to carry Lipid A (the toxic, fatty acid-rich portion of LPS) into the brain. Our data in Fig 5 support this model, which is physiologically feasible as an estimated 20–30% of albumin normally transitions into the interstitial space [44].

Albumin is considered the safest colloid [45]. Our findings may explain the contraindication of intravenous albumin in traumatic brain injury (TBI) patients, which is associated with significantly increased mortality [46]. We propose a mechanism: TBI disrupts intestinal barrier integrity, leading to the translocation of commensal gut bacteria into systemic circulation [47]. Therefore, we propose that LPS derived from gut bacteria, particularly Gram-negative bacteria, enters the bloodstream and binds to intravenous infused albumin. Under pathological conditions such as inflammation, BBB permeability to foreign substances increases [48], thereby allowing the LPS-albumin complex to enter the brain.

Plasma exchange with albumin replacement has been an effective approach for combating AD [49,50]. The goal of this treatment is to absorb more Abeta in the blood and to lower the Abeta level in cerebrospinal fluid (CSF). Because albumin can also interact with LPS, it is possible that plasma exchange with albumin replacement may also remove a part of the LPS in the blood, and that the new albumin can absorb more LPS, as LPS levels has been found elevated in AD brains [51].

Albumin is the most abundant protein in the CSF, accounting for approximately 60% of total protein, compared to only 49% in plasma [52]. Furthermore, certain brain regions, including the circumventricular organs, are not protected by the BBB [53,54]. Given the high percentage of albumin in the CSF, once the CSF is infected by Gram-negative bacteria, this high albumin level may greatly increase the entry of LPS into the brain parenchyma.

In the pre-treatment of conventional Co-IP experiments, protein denaturation during SDS-PAGE disrupts non-covalently bound protein complexes and linearizes proteins. However, studies have shown that sugar chains can still associate with proteins after SDS-PAGE treatment [55]. Therefore, our study indicates that LPS interacts with albumin. Specifically, albumin likely binds to the LPS Core component of LPS, which is why we detected the LPS Core band in the Co-IP experiments.

However, our study still has the following limitations. First, it does not account for other molecules with high binding affinity for BSA, such as the lipofuscin-related product N-retinylidene-N-retinylethanolamine (A2E). It has been reported that the binding of A2E to BSA enhances the oxidative photoreactivity of A2E [56], suggesting that such molecules may produce effects distinct from those of LPS. Furthermore, we did not establish individual dose–response curves for each drug; therefore, drug effect ratios could not be compared based on equivalent doses. Future studies are needed to validate the function of the BSA–LPS complex in other models and to conduct independent dose–response relationship studies for each drug.

## Supporting information

S1 Fig The impact of BSA on AF. (A, B) AF and DAPI fluorescence were observed on brain tissue sections of mice after DAPI staining, 2 weeks after intraperitoneal administration of BSA. Scale bar: 20 µm.
(TIF)

S2 Fig The impact of BSA on vascular cell permeability. (A, B) Immunofluorescence staining for CD31 and CD13 was performed on tissue samples 10 min after intravenous injection of PI. Scale bar: 20 µm.
(TIF)

S3 Fig Effects of BSA on the BBB integrity. (A) Results of intravenously injected HRP circulating for 10 min 24 h after BSA administration. (B) Staining results of neuronal IgG in the samples 24 h after BSA administration. (C) Staining results of endogenous peroxidase in the samples 24 h after BSA administration. Scale bar: 50 µm.
(TIF)

S4 Fig Effects of BSA on CD68. Immunofluorescence staining for CD68 was performed on brain tissue sections of mice, 24 h after intraperitoneal administration of BSA, and the corresponding DAPI nuclear staining images were presented. Scale bar: 20 µm.
(TIF)
